# TPL-2 Inhibits IFN-β Expression via an ERK1/2-TCF-FOS Axis in TLR4-Stimulated Macrophages

**DOI:** 10.4049/jimmunol.2100213

**Published:** 2022-02-15

**Authors:** Louise Blair, Michael J. Pattison, Probir Chakravarty, Stamatia Papoutsopoulou, Latifa Bakiri, Erwin F. Wagner, Stephen Smale, Steven C. Ley

**Affiliations:** * Immune Cell Signalling Laboratory, The Francis Crick Institute, London, United Kingdom;; † Bioinformatics and Biostatistics Technology Platform, The Francis Crick Institute, London, United Kingdom;; ‡ Laboratory of Genes and Disease, Department of Laboratory Medicine, Medical University of Vienna, Vienna, Austria;; § Laboratory of Genes and Disease, Department of Dermatology, Medical University of Vienna, Vienna, Austria;; ¶ Department of Microbiology, Immunology and Molecular Genetics, University of California, Los Angeles, CA; and; ‖ Department of Immunology and Inflammation, Imperial College London, London, United Kingdom

## Abstract

TPL-2 kinase plays an important role in innate immunity, activating ERK1/2 MAPKs in myeloid cells following TLR stimulation. We investigated how TPL-2 controls transcription in TLR4-stimulated mouse macrophages. TPL-2 activation of ERK1/2 regulated expression of genes encoding transcription factors, cytokines, chemokines, and signaling regulators. Bioinformatics analysis of gene clusters most rapidly induced by TPL-2 suggested that their transcription was mediated by the ternary complex factor (TCF) and FOS transcription factor families. Consistently, TPL-2 induced ERK1/2 phosphorylation of the ELK1 TCF and the expression of TCF target genes. Furthermore, transcriptomic analysis of TCF-deficient macrophages demonstrated that TCFs mediate approximately half of the transcriptional output of TPL-2 signaling, partially via induced expression of secondary transcription factors. TPL-2 signaling and TCFs were required for maximal TLR4-induced FOS expression. Comparative analysis of the transcriptome of TLR4-stimulated *Fos*^−/−^ macrophages indicated that TPL-2 regulated a significant fraction of genes by controlling FOS expression levels. A key function of this ERK1/2-TCF-FOS pathway was to mediate TPL-2 suppression of type I IFN signaling, which is essential for host resistance against intracellular bacterial infection.

## Key Points

- TPL-2 activation of ERK1/2 regulates gene expression in TLR-stimulated macrophages.
- TPL-2 regulates transcription via ERK1/2 phosphorylation of ternary complex factors.
- TPL-2 inhibits *Ifnb1* transcription via ternary complex factor–induced *Fos* mRNA expression.

## Introduction

Macrophages are a critical cellular component of the immune system, responding rapidly to pathogens to initiate innate immune responses (1). Following infection, macrophages are stimulated by invariant pathogen molecules, termed pathogen-associated molecular patterns, via pattern recognition receptors. For example, LPS from Gram-negative bacterial cell walls binds to the pattern recognition receptor TLR4, expressed at the plasma membrane and on endosomes in macrophages (2). TLR stimulation of macrophages triggers the coordinate activation of several intracellular signaling pathways and the expression of multiple genes, which include the cytokines and chemokines responsible for promoting an inflammatory response and coordinating an effective adaptive immune response (3).

TLR stimulation results in the activation of the IκB kinase (IKK) complex, leading to the nuclear translocation of NF-κB transcription factor dimers, and the activation of classical MAPKs ERKs 1 and 2 (ERK1/2), JNKs 1 and 2 (JNK1/2), and p38α (4, 5). NF-κB and MAPK activation together promote expression of multiple proinflammatory cytokines. Stimulation of certain TLRs, including TLR3 and TLR4, also activates TANK binding kinase 1 (TBK1), which phosphorylates IFN regulatory factors (IRFs) and drives the expression of type I IFNs.

TPL-2 (MAP3K8) is a MAPK kinase kinase that mediates activation of ERK1/2 and p38α MAPKs downstream of TLRs in macrophages, neutrophils, and dendritic cells (DCs) (6–8). In unstimulated cells, TPL-2 exists in a ternary complex with ABIN-2 and NF-κB1 p105. Following TLR stimulation, IKK2 phosphorylates p105, leading to p105 degradation by the proteasome, releasing TPL-2 from p105-mediated inhibition. IKK2 also directly phosphorylates TPL-2 Ser^400^, inducing 14-3-3 dimer binding to the TPL-2 C terminus, increasing TPL-2 catalytic activity (9). Together, these two IKK2-regulated steps promote TPL-2 to phosphorylate its substrates, MAPK kinase (MKK)1/2 (*Map2k1/2*) and MKK3/6 (*Map2k3/6*) (8, 10–12). MKK1/2 in turn phosphorylate and activate ERK1/2, whereas MKK3/6 phosphorylate and activate p38 MAPKs. Although MKK3/6 activation is dependent on TPL-2 following TLR4 stimulation, the p38α-activating kinase MKK4 is activated independently of TPL-2. Consequently, TLR4 activation of p38α in macrophages is only marginally reduced by the absence of TPL-2 signaling, whereas ERK1/2 activation is blocked.

Analyses of *Tpl2*^−/−^ macrophages indicate that TPL-2 stimulates the expression of a number of inflammatory mediators following LPS stimulation of macrophages, including IL-1β, TNF, CCL2, CXCL2, and COX2 (13–15). Pharmacological inhibition of MKK1/2 inhibition phenocopies the effects of TPL-2 deficiency, suggesting that TPL-2 controls TLR4 induction of these genes via ERK1/2 activation. Consistent with these inhibitory effects on proinflammatory gene expression, TPL-2 deficiency reduces inflammation in a number of preclinical disease models. *Tpl2*^−/−^ mice are protected from LPS-induced septic shock, TNF-induced inflammatory bowel disease, experimental autoimmune encephalomyelitis, acute pancreatitis, and liver injury (14–18). TPL-2 is consequently considered a potential anti-inflammatory drug target, particularly for inflammatory bowel disease, the development of which is linked genetically to *MAP3K8* (19).

TPL-2 signaling also has inhibitory effects on gene expression. For example, we have previously shown that TPL-2 activation of ERK1/2 inhibits IFN-β transcription in LPS-stimulated macrophages and DCs (20). Subsequent experiments underlined the importance of this negative regulation, demonstrating that TPL-2 inhibition of type I IFN signaling is essential for effective innate immune responses to *Mycobacteria tuberculosis* and *Listeria monocytogenes* (21). Consistent with this, type I IFNs are known to have a detrimental effect on immune responses to these bacteria, abrogating IFN-γ–dependent host-protective immune responses via downregulation of the IFN-γ receptor, while promoting the production of the anti-inflammatory cytokine IL-10 (22).

The mechanisms by which TPL-2 signaling alters transcription is not known. In this study, we used RNA sequencing (RNA-seq) to demonstrate that ternary complex factors (TCFs) have an important role in mediating the effects of the TPL-2 MAPK pathway on gene expression in TLR4-stimulated macrophages. TCFs are direct targets of the RAF–ERK1/2 MAP kinase pathway regulating immediate early gene transcription and a gene expression program that controls proliferation in serum-stimulated fibroblasts (23, 24). The results of this study demonstrate that TCFs also control gene expression in TLR-stimulated primary mouse macrophages downstream of the TPL-2–ERK1/2 MAPK pathway, suggesting an important regulatory role for this family of transcription factors in innate immune responses.

## Materials and Methods

### Mouse strains

Mouse strains were bred in a specific-pathogen-free environment at the Francis Crick Institute (London, U.K.), and all experiments were done in accordance with regulations of the U.K. Home Office. *Nfkb1*^SSAA/SSAA^, *Fos^fl/fl^*, *Elk1*^−/−^*Elk4*^−/−^, *LysM-Cre*, and *Map3k8*^D270A/D270A^ mouse strains have been described previously (11, 25–27) and were all fully backcrossed onto a C57BL/6 background.

### Abs used

Abs against ERK1/2, phospho-ERK1/2 (Thr^202^/Tyr^204^), FOS, ATF2, and JUN were purchased from Cell Signaling Technology. Phospho-ELK1 (Ser^389^) Ab was supplied by Invitrogen. Phospho-IRF3 (Ser^385^) Ab was purchased from Thermo Fisher Scientific, and phospho-TBK1 (Ser^172^) Ab was purchased from BD Biosciences. ELK1 Ab was obtained from Abcam.

### In vitro generation and stimulation of macrophages

Bone marrow-derived macrophages (BMDMs) were prepared as described previously (10). For experiments, harvested BMDMs were replated in Nunc tissue culture dishes (12- or 6-well plates, 0.5 × 10^6^ cells/ml) in RPMI 1640 medium (Sigma) supplemented with 1% FBS, antibiotics, and 50 μM 2-ME. After overnight culture, LPS (*Salmonella* Enterica serovar Minnesota R595; Alexis Biochemicals) was added at a final concentration of 100 ng/ml. Control cells were left untreated (time 0). For experiments in which ERK1/2 kinase activation was blocked pharmacologically, cells were preincubated with 100 nM PD0325901 (MKK1/2 inhibitor; purchased from MRC-PPU Reagents, Dundee University) or ribosomal S6 kinase (RSK)i47 (RSK inhibitor; purchased from MRC-PPU Reagents, Dundee University) for 10 min prior to LPS stimulation.

### RNA-seq

RNA was isolated using the RNeasy kit (QIAGEN). Biological replicate libraries were prepared using the TruSeq RNA library prep kit (Illumina) and were single-end sequenced on the Illumina HiSeq 2500 platform. Sequencing yield was typically ∼25 million strand-specific reads. The RSEM package (version 1.2.31) (28) in conjunction with the STAR alignment algorithm (version 2.5.2a) (29) was used for the mapping and subsequent gene-level counting of the sequenced reads with respect to mm10 RefSeq genes downloaded from the UCSC Table Browser 15 on February 19, 2016 (30). Differential expression analysis was performed with the DESeq2 package (version 1.10.1) (31) within the R programming environment (version 3.2.3) (32). Genes with a false discover rate <0.05 were judged to be differentially expressed.

### Quantitative RT-PCR

RNA was isolated using the RNeasy kit (Qiagen). Five hundred to 1000 ng of RNA was reverse transcribed using the SuperScript VILO cDNA synthesis kit (Invitrogen). mRNA expression was measured using TaqMan probes and TaqMan gene expression master mix (Applied Biosystems) on QuantStudio 3 and 5 real-time PCR systems (Applied Biosystems). Data were normalized to hypoxanthine phosphoribosyltransferase using the cycle threshold (ΔΔCt) method.

### IFN-β ELISA

Secreted IFN-β in tissue culture supernatant was quantified using the VeriKine mouse IFN-β ELISA kit (PBL Assay Science) as per the manufacturer’s instructions.

### In vivo challenge with LPS

Fos knockout (FosKO) mice and LysM-Cre controls, aged between 10 and 14 wk, were injected i.p. with 5 mg/kg LPS (*Escherichia coli* O111:B4, Sigma-Aldrich). After 4 h, mice were sacrificed via cervical dislocation and blood was extracted from the heart using a 25G needle and 0.3-ml syringe. Blood was left to coagulate for 30 min and then centrifuged at 17,000 × *g* for 1 min. Serum was stored at −80°C until the assay for IFN-β by ELISA.

### Chromatin immunoprecipitation

Chromatin immunoprecipitation (ChIP) assays were performed as described previously (23). Briefly, 2- × 15-cm plates of 14 × 10^6^ cells were cross-linked. Samples were sonicated using 10 cycles of 30 s on, 30 s off. Samples were precleared for 1 h prior to overnight incubation with Abs. Samples were washed and then crosslinks were reversed overnight. Samples were then purified using a PCR purification kit (QIAGEN) before analysis using quantitative PCR. Percentage input was calculated and then data were normalized and pooled from three or four experiments. The following Abs were used: Rbp1 NTD (D8L4Y) (Cell Signaling Technology), ELK1 (E277) (Abcam), and SRF (1681-1-AP, Lot 1) (Proteintech).

### TransAM ELISA assay

For ELISA of NF-κB and AP-1 activity, nuclear extracts were prepared using a commercial kit (Active Motif). Five micrograms of extract per point was assayed in duplicate, using a TransAm NF-κB or AP-1 kit (Active Motif), monitoring RelA and FOS binding, respectively.

### Immunoblotting

Immunoblotting of cell lysates was carried out as described previously (33).

### Statistical analysis

Analyses of quantitative PCR and ELISA data were performed using GraphPad software (GraphPad Software, San Diego, CA). Data were compared using one-way or two-way ANOVA as appropriate. A *p* value <0.05 or <0.001 was considered significant (*p < 0.05, **p < 0.01). Error bars represent SEM.

### Data availability

All of the raw RNA-seq datasets of this study have been deposited in Gene Expression Omnibus under accession numbers GSE178921 (https://www.ncbi.nlm.nih.gov/geo/query/acc.cgi?acc=GSE178921) and GSE116220 (https://www.ncbi.nlm.nih.gov/geo/query/acc.cgi?acc=GSE116220).

## Results

### Regulation of gene expression in LPS-stimulated macrophages by TPL-2 catalytic activity

**FIGURE 1.**
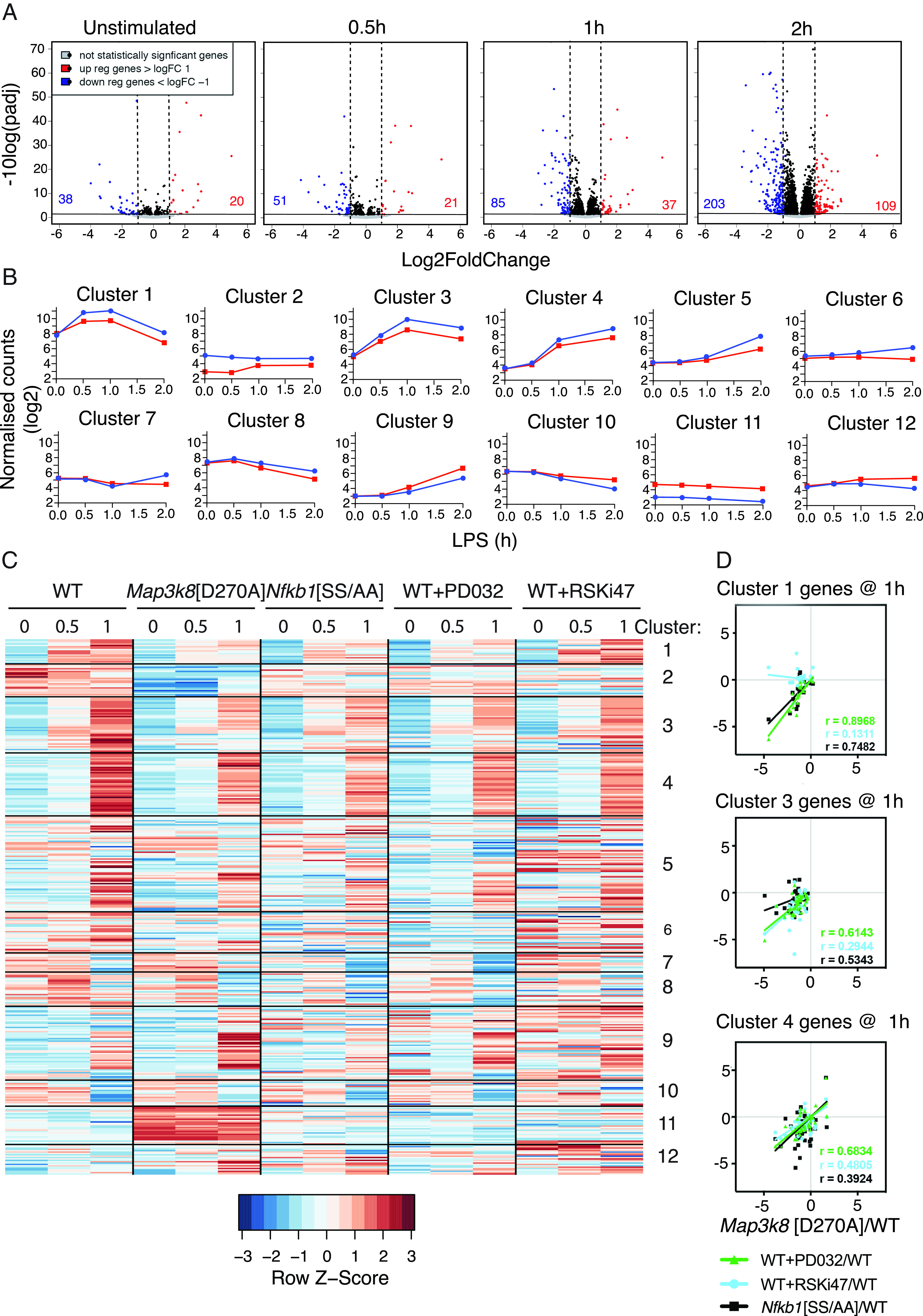
TPL-2 kinase activity induces and represses gene expression in LPS-stimulated macrophages. (**A**) Volcano plots of log_2_-transformed expression values and statistical significance of RNA-seq analyses of WT and *Map3k8*[D270A] macrophages stimulated with LPS for the indicated times. Genes significantly upregulated or downregulated (>2-fold change and false discovery rate < 0.05) are indicated by red or blue dots, respectively. (**B**) Log_2_-transformed normalized counts for WT and *Map3k8*[D270A] BMDMs for each cluster identified by k-means analysis. WT, blue; *Map3k8*[D270A], red. (**C**) Heatmap showing row-normalized expression for WT, *Map3k8*[D270A], *Nfkb1*[SS/AA], WT+PD0325901, and WT+RSKi47 macrophages for genes regulated by TPL-2 across a 1-h time course. Genes are presented in the clusters from the k-means analysis of TPL-2–regulated genes. (**D**) Log_2_ fold changes for *Map3k8*[D270A] compared with WT were taken for each gene in specific clusters and plotted against the equivalent fold changes of *Nfkb1*[SS/AA], WT+PD0325901, and WT+RSKi47 compared with WT. Each dot indicates a gene, solid lines indicate the linear regression, and *r* values were calculated using Spearman rank correlation.

Wild-type (WT) BMDMs and *Map3k8*^D270A/D270A^ BMDMs, which express kinase-inactive TPL-2^D270A^ (16), were stimulated with LPS during a time course (0.5, 1, and 2 h) and gene expression was analyzed by RNA-seq. TPL-2 kinase activity was required for maximal expression of 131 genes during the time course (≥2-fold reduction in *Map3k8*^D270A/D270A^ cells compared with WT at any time point; (Fig. 1A, Supplemental Fig. 1A, Supplemental Table I). The expression of 278 genes was suppressed by TPL-2 catalytic activity (≥2-fold increase in *Map3k8*^D270A/D270A^ cells compared with WT at any time point; (Fig. 1A, Supplemental Fig. 1A, Supplemental Table I). The number of genes positively and negatively regulated by TPL-2 catalytic activity increased as the time of LPS stimulation increased (Fig. 1A).

To gain some insight into the potential impact of TPL-2 signaling on the biological response of macrophages to LPS stimulation, the genes regulated by TPL-2 catalytic activity were analyzed for process and pathway enrichment using the MetaCore program (Supplemental Fig. 1B). Immune response pathways, in particular cytokine and growth factor signaling via MAPK, NF-κB, and JAK-STAT signaling pathways, and damage-/injury-associated pathways were highly enriched. Similarly, immune response, inflammation, and signaling processes were enriched. These results are consistent with the known importance of TPL-2 in innate immunity, adaptive immunity, and autoimmune inflammation, based on experiments with *Map3k8*^−/−^ mice (7).

TPL-2–regulated genes were grouped into 12 distinct kinetic groups by k-means cluster analysis (Fig. 1B). Because TPL-2 signaling is rapidly and transiently induced following LPS stimulation of macrophages (33), we focused on clusters 1, 3, and 4, which contained genes that were clearly induced at the early time points following LPS stimulation (Supplemental Fig. 2). Cluster 1 contained the genes most rapidly induced by LPS stimulation and these were strongly reduced by *Map3k8*^D270A^ mutation. RNA-seq determined that LPS induction of cluster 1 genes was also significantly reduced by *Nfkb1*^SSAA^ mutation (11), which prevents signal-induced proteolysis of p105 and consequently prevents TPL-2 activation of MKK1/2 and their substrates ERK1/2 (Fig. 1C). Similarly, pharmacological inhibition of ERK1/2 activation in WT cells with PD0325901 MKK1/2 inhibitor reduced the expression of cluster 1 genes (Fig. 1C). Pharmacological inhibition of RSK1/2 with RSKi47 (34) did not alter LPS-induced expression of mRNAs encoding cluster 1 genes (Fig. 1C), indicating that TPL-2 did not induce cluster 1 gene expression as a consequence of ERK1/2-mediated activation of RSK1/2. Genes in clusters 3 and 4 had a delayed induction compared with cluster 1, but their expression was similarly reduced by *Map3k8*^D270A^ and *Nfkb1*^SSAA^ mutation, as well as by PD0325901 treatment. Inhibition of RSK1/2 activity with RSKi47 also reduced expression of a fraction of cluster 3 and 4 genes. Interestingly, suppressed expression of cluster 11 genes by TPL-2 catalytic activity was not phenocopied by MKK1/2 inhibition, suggesting that TPL-2 regulated the constitutive expression of a subset of genes independently of ERK1/2 activation.

The overlaps between the effects of *Map3k8*^D270A^ mutation and *Nfkb1*^SSAA^ mutation or PD0325091 treatment on the expression of individual genes in clusters 1, 3, and 4 were compared quantitatively. At 1 h of LPS stimulation, the expression values in cluster 1 correlated well between *Map3k8*^D270A/D270A^ and *Nfkb1*^SSAA/SSAA^ cells (*r* = 0.748) and between *Map3k8*^D270A/D270A^ cells and WT cells + PD0325901 (*r* = 0.897) (Fig. 1D). Similar comparisons in the expression values for clusters 3 and 4 were also well correlated (Fig. 1D). The RNA-seq experiments therefore supported the hypothesis that TPL-2 catalytic activity promoted the expression of rapidly induced genes in clusters 1, 3, and 4 via activation of ERK1/2, which was dependent on IKK-induced proteolysis of NF-κB1 p105, an essential step for TPL-2 activation of ERK1/2 following LPS stimulation (11). The expression values in clusters 1 and 3 only weakly or moderately correlated between *Map3k8*^D270A/D270A^ cells and WT cells + RSKi47, suggesting that RSKs did not play a major role in regulating the expression of these gene clusters. However, the effects of *Map3k8*^D270A^ mutation and RSKi47 correlated more strongly for cluster 4 genes, suggesting that TPL-2 activation of RSKs contributed to the expression of these genes.

### TPL-2 catalytic activity induces the expression of TCF target genes

**FIGURE 2.**
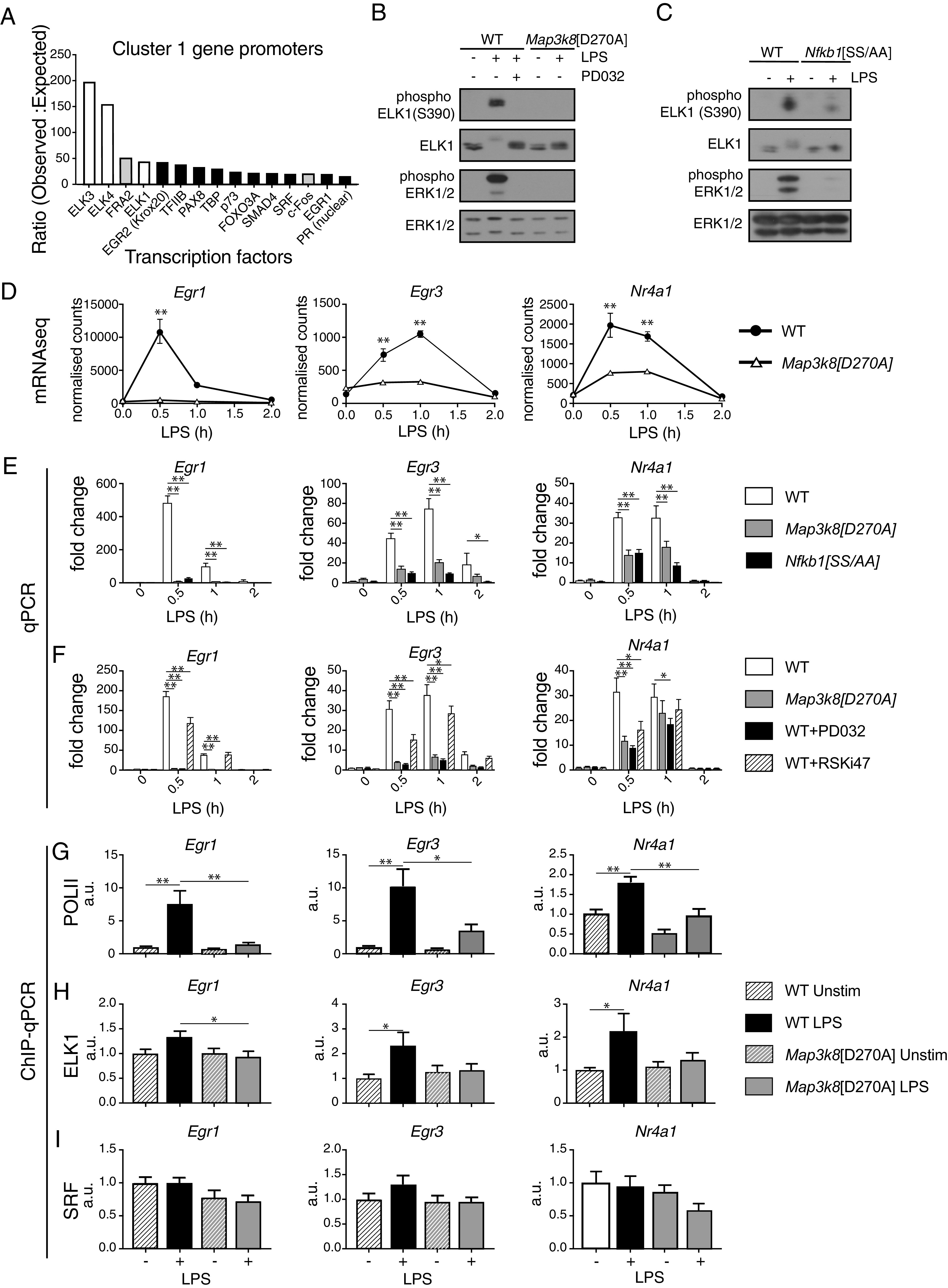
TPL-2 regulates ELK1 phosphorylation and TCF target gene expression. (**A**) Genes in cluster 1 were analyzed for transcription factor enrichment using the MetaCore program. The top 15 enriched transcription factors are shown ranked by the ratio of observed sites to expected sites in the cluster 1 gene promoters. TCF family transcription factors, white; Fos family transcription factors, light gray. (**B**) WT and *Map3k8*[D270A] macrophages stimulated with LPS for 15 min. PD0325901 was added to the indicated WT cultures to block ERK1/2 activation. Total cell lysates were immunoblotted for the antigens shown. (**C**) WT and *Nfkb1*[SS/AA] macrophages stimulated with LPS for 15 min. Total cell lysates were immunoblotted for the indicated antigens. Experiments in (B) and (C) are representative of three similar experiments. (**D**) Normalized counts from the RNA-seq analysis of WT and *Map3k8*[D270A] macrophages unstimulated or stimulated with LPS for the indicated times are shown for *Egr1*, *Egr3*, and *Nr4a1*. (**E**) WT, *Map3k8*[D270A], and *Nfkb1*[SS/AA] macrophages stimulated with LPS during a 2-h time course. *Egr1*, *Egr3*, and *Nr4a1* mRNA expression levels were analyzed by qRT-PCR. (**F**) WT, *Map3k8*[D270A], WT+PD0325901, and WT+RSKi47 macrophages stimulated with LPS during a 2-h time course. *Egr1*, *Egr3*, and *Nr4a1* mRNA expression analyzed by qRT-PCR. Results in (E) and (F) are representative of three similar experiments. (**G**) WT and *Map3k8*[D270A] macrophages were stimulated with LPS for 30 min or left untreated. Chromatin immunoprecipitations (ChIPs) were performed with (G) POLII, (**H**) ELK1, and (**I**) SRF Abs. ChIP signals relative to input for TCF binding regions of the *Egr1*, *Egr3*, and *Nr4a1* promoters are shown. Results from three separate experiments were combined. *p ≤ 0.05, **p ≤ 0.01.

Studies in fibroblasts have demonstrated that ERK1/2 directly phosphorylates ∼50 transcription factors following growth factor receptor stimulation, including ATF2, C/EBPβ, ELK1, ERF, c-FOS, HIF1α, SP1, and c-JUN (35). The mechanisms by which TPL-2 activation of ERK1/2 regulates transcription in macrophages are not understood. We used a bioinformatics approach to investigate this question, focusing on TPL-2–regulated genes in cluster 1 that were most rapidly induced by LPS stimulation and therefore likely to be direct targets of the TPL-2–ERK1/2 signaling pathway. Analysis of these genes using the MetaCore database revealed that ELK3, ELK4, and ELK1 were among the four most enriched transcription factors (based on a ratio of observed/expected hits) (Fig. 2A). ELK3, ELK4, and ELK1 belong to the TCF subfamily of ETS family transcription factors (36).

Experiments in fibroblasts have demonstrated that TCFs bind cooperatively with serum response factor (SRF) to serum response elements in the promoters of immediate early genes (37). Mitogenic stimulation activates the Ras-Raf MAPK pathway, inducing ERK1/2 phosphorylation of C-terminal transactivation domains of TCFs, which triggers their transcriptional activation and immediate early gene expression (38). Immunoblotting of macrophage cell lysates demonstrated that LPS stimulation induced ELK1 phosphorylation at Ser^390^, an ERK1/2 phosphorylation site, and this was blocked by *Map3k8*^D270A^ mutation (Fig. 2B). LPS-induced ELK1 Ser^390^ phosphorylation was also prevented by PD0325901 MKK1/2 inhibitor treatment of WT cells and by *Nfkb1*^SSAA^ mutation (Fig. 2B, 2C). These data indicated that TPL-2 signaling induced C-terminal ELK1 phosphorylation by ERK1/2, downstream of IKK-induced NF-κB1 p105 proteolysis.

Analysis of the RNA-seq data revealed that *Map3k8*^D270A^ mutation significantly reduced the mRNA abundance of the TCF-regulated immediate early genes *Egr1*, *Egr3*, and *Nr4A1* following LPS stimulation of macrophages (Fig. 2D), which was confirmed by quantitative RT-PCR (qRT-PCR) (Fig. 2E). *Nfkb1*^SSAA^ mutation and treatment of WT cells with PD0325901 also reduced the LPS-induced expression of *Egr1*, *Egr3*, and *Nr4A1* mRNAs in macrophages (Fig. 2E, 2F). Primary transcript qRT-PCR demonstrated that *Map3k8*^D270A^ mutation reduced mRNA levels of these genes by impairing their transcription (Supplemental Fig. 3A). Consistent with this, ChIP experiments showed that LPS stimulation induced recruitment of RNA polymerase II to the promoters of *Egr1*, *Egr3*, and *Nr4A1*, which was reduced by *Map3k8*^D270A^ mutation (Fig. 2G). Binding of ELK1 to the *Egr1, Egr3*, and *Nr4a1* promoters was fractionally increased in LPS-stimulated WT cells, and this increase was blocked by *Tpl2*^D270A^ mutation (Fig. 2H). In contrast, binding of SRF was constitutive to the *Egr1, Egr3*, and *Nr4a1* promoters and unaffected by *Map3k8*^D270A^ mutation (Fig. 2I). Taken together, these results indicated that TLR4 activation of the IKK2–NF-κB1 p105–TPL-2–ERK1/2 MAPK pathway promoted TCF phosphorylation, thereby inducing transcription of TCF/SRF-regulated immediate-early genes.

### ELK1/ELK4 regulate nearly half of TPL-2–dependent genes in LPS-stimulated macrophages

**FIGURE 3.**
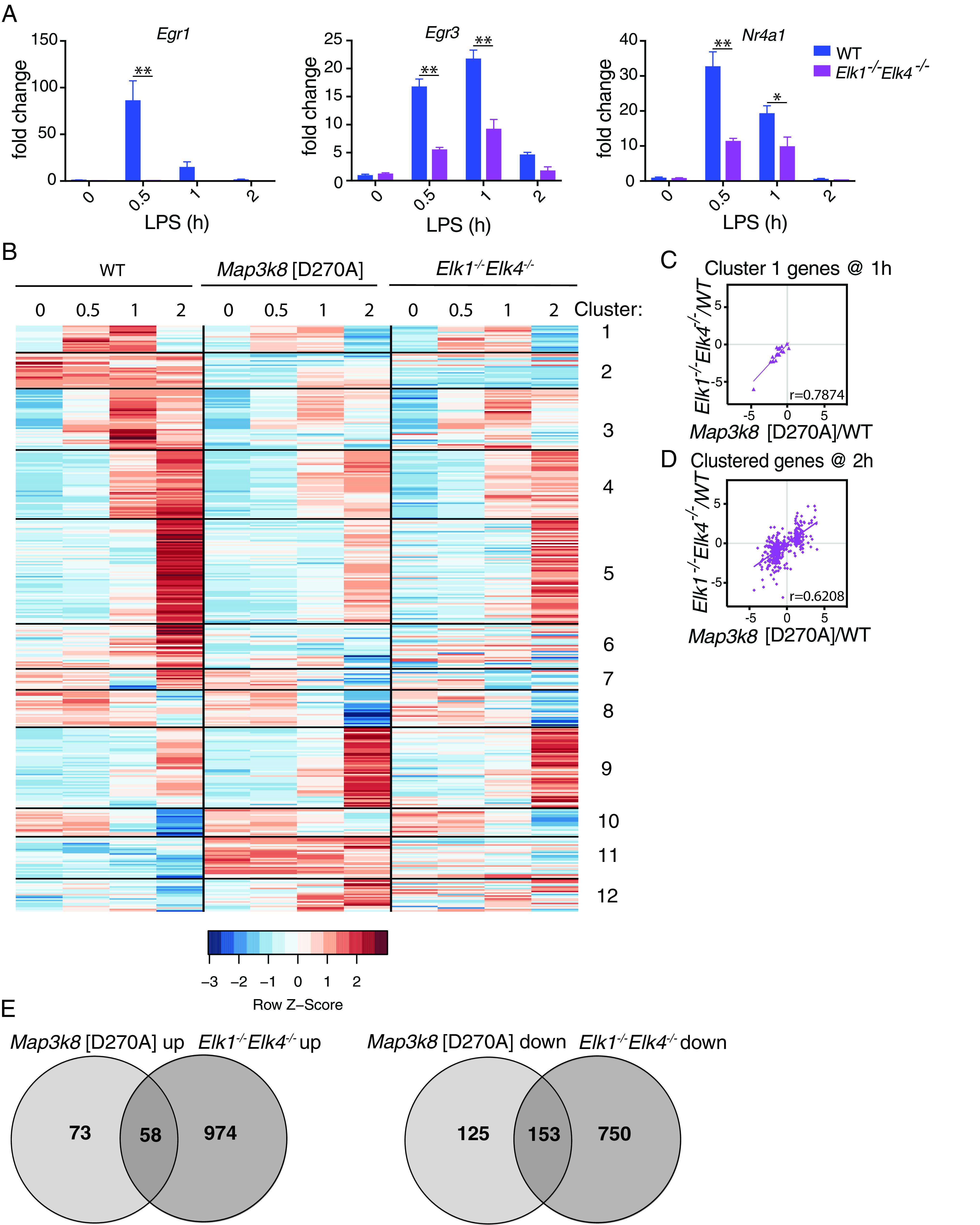
TPL-2 regulates gene transcription via ELK1 and ELK4. (**A**) WT and *Elk1*^−/−^*Elk4*^−/−^ macrophages were stimulated with LPS for a 2-h time course. *Egr1*, *Egr3*, and *Nr4a1* mRNA levels were determined by qRT-PCR. Results are representative of three similar experiments. (**B**) Heatmap showing row-normalized expression for WT, *Map3k8*[D270A] (as in (Fig. 1A), and *Elk1*^−/−^*Elk4*^−/−^ BMDMs for clustered genes regulated by TPL-2 during a 2-h time course. (**C** and **D**) Log_2_ fold changes for *Map3k8*[D270A]/WT for each gene in cluster 1 at 1 h (C) or all clustered genes at 2 h (D), plotted against the equivalent fold changes of *Elk1*^−/−^*Elk4*^−/−^/WT comparison. Each dot indicates a gene, solid lines indicate the linear regression, and *r* values were calculated using Spearman rank correlation. (**E**) Venn diagrams show overlaps of genes upregulated or downregulated (>2-fold) by *Map3k8*[D270A] mutation and ELK1/ELK4 deficiency, calculated from *Map3k8*[D270A]/WT and *Elk1*^−/−^*Elk4*^−/−^/WT RNA-seq comparisons. *p ≤ 0.05, **p ≤ 0.01.

The roles of TCFs in the regulation of gene expression have been intensively studied in serum-stimulated fibroblasts (23, 39). However, the contribution of TCFs to gene expression in innate immune responses is not known. ELK1 and ELK4 act redundantly and their knockout phenotypes are distinct from those of ELK3 (27, 40). We therefore analyzed TLR4-induced gene expression in macrophages generated from *Elk1*^−/−^ *Elk4*^−/−^ mice. As expected, qRT-PCR demonstrated that LPS-induced expression of *Egr1*, *Egr3*, and *Nr4a1* mRNAs was significantly reduced in *Elk1*^−/−^ *Elk4*^−/−^ macrophages compared with WT cells (Fig. 3A).

RNA-seq was used to examine the global effect of ELK1/ELK4 deficiency on LPS-induced gene expression in BMDMs (Supplemental Table I). Heatmap analysis showed that *Map3k8*^D270A^ mutation and ELK1/ELK4 deficiency had overlapping effects on gene expression (Fig. 3B). As expected, cluster 1 gene expression showed a strong correlation between *Map3k8*^D270A/D270A^ and *Elk1*^−/−^*Elk4*^−/−^ BMDMs at 1 h (*r* = 0.79) (Fig. 3C). Strikingly, there was also a strong overall correlation at the 2-h time point comparing genes in all clusters between *Map3k8*^D270A/D270A^ and *Elk1*^−/−^*Elk4*^−/−^ macrophages (*r* = 0.62) (Fig. 3D). Of the 278 genes whose expression was decreased by *Map3k8*^D270A^ mutation (≥2-fold) during the 2-h time course, 55% (153/278) were also downregulated (≥2-fold) in LPS-stimulated *Elk1*^−/−^*Elk4*^−/−^ BMDMs compared with WT cells (Fig. 3E). Furthermore, 44% (58/131) of genes whose expression was increased (≥2-fold) by *Map3k8*^D270A^ mutation were also upregulated (≥2-fold) in LPS-stimulated *Elk1*^−/−^*Elk4*^−/−^ BMDMs compared WT cells. These results indicate that TCFs mediate a major transcriptional output of TPL-2 signaling in TLR4-stimulated macrophages.

In total, 211 genes were found to be >2-fold coregulated by TPL-2 and TCFs. Analysis of these genes using the MetaCore program revealed an enrichment for processes involving Th17 cytokines, chemotaxis, signaling, and proliferation (Supplemental Fig. 3B). The pathway enrichment analysis demonstrated the involvement of these genes in a range of immune and inflammation pathways, including IL-1 signaling and inflammatory responses associated with asthmatic airway fibroblasts. This analysis suggests that TCFs activated by the TPL-2–ERK1/2 MAPK pathway in macrophages can regulate genes that control a number of aspects of innate immune responses.

### Regulation of *Fos* family genes by the TPL-2–ERK1/2–TCF signaling pathway

Experiments with TCF-deficient fibroblasts have shown that >60% of 12-*O*-tetradecanoylphorbol-13-acetate (TPA)–induced gene transcription is mediated via ERK1/2 activation of TCFs (23). Direct TCF-SRF targets in these cells encompass 54 different transcription factors, suggesting that TCFs control transcription of many genes indirectly via induced expression of these downstream transcription factors, which include members of the Egr, AP-1, Ets, and NF-κB families.

MetaCore analysis of TPL-2–regulated genes in gene clusters 1, 3, and 4 in LPS-stimulated macrophages suggested a role for AP-1 transcription factors in mediating the transcriptional output of TLP-2 (Fig. 2A, Supplemental Fig. 4A, 4B). The AP-1 family is composed of 15 polypeptides, which include the FOS, JUN, and ATF subfamilies (41), which bind to DNA as obligate dimers via conserved bZIP domains. Binding sites for all FOS family members (FOS, FRA-1, FRA-2, and FOS-B) were enriched across the promoters of TPL-2–regulated genes in clusters 1 and 4. In addition, ATF2 and ATF3 binding sites were enriched in clusters 4 and 3 gene promoters, respectively, and JUN, JUN-B, and JUN-D binding sites were enriched in the promoters of cluster 4 genes.

Analysis of the *Map3k8*^D270A/D270A^/WT RNA-seq dataset indicated that TPL-2 signaling promoted the LPS-induced mRNA expression of all *Fos* and *Jun* family members (Supplemental Fig. 4C). *Map3k8*^D270A^ mutation significantly reduced *Fos* and *FosB* mRNA levels at 0.5 and 1 h of LPS stimulation, whereas *Fra1* (*Fosl1*) and *Fra2* (*Fosl2*) were downregulated only at later time points. The expression of *Jund* mRNA was reduced by *Map3k8*^D270A^ mutation at all time points, including unstimulated cells, whereas *Jun* and *JunB* mRNA levels were only reduced following LPS stimulation. The ATF family of genes were less affected by *Map3k8*^D270A^ mutation. These data raised the possibility that TPL-2 activation of TCFs regulated AP-1–mediated gene expression by modulating the transcription of *Fos* and *Jun* genes.

**FIGURE 4.**
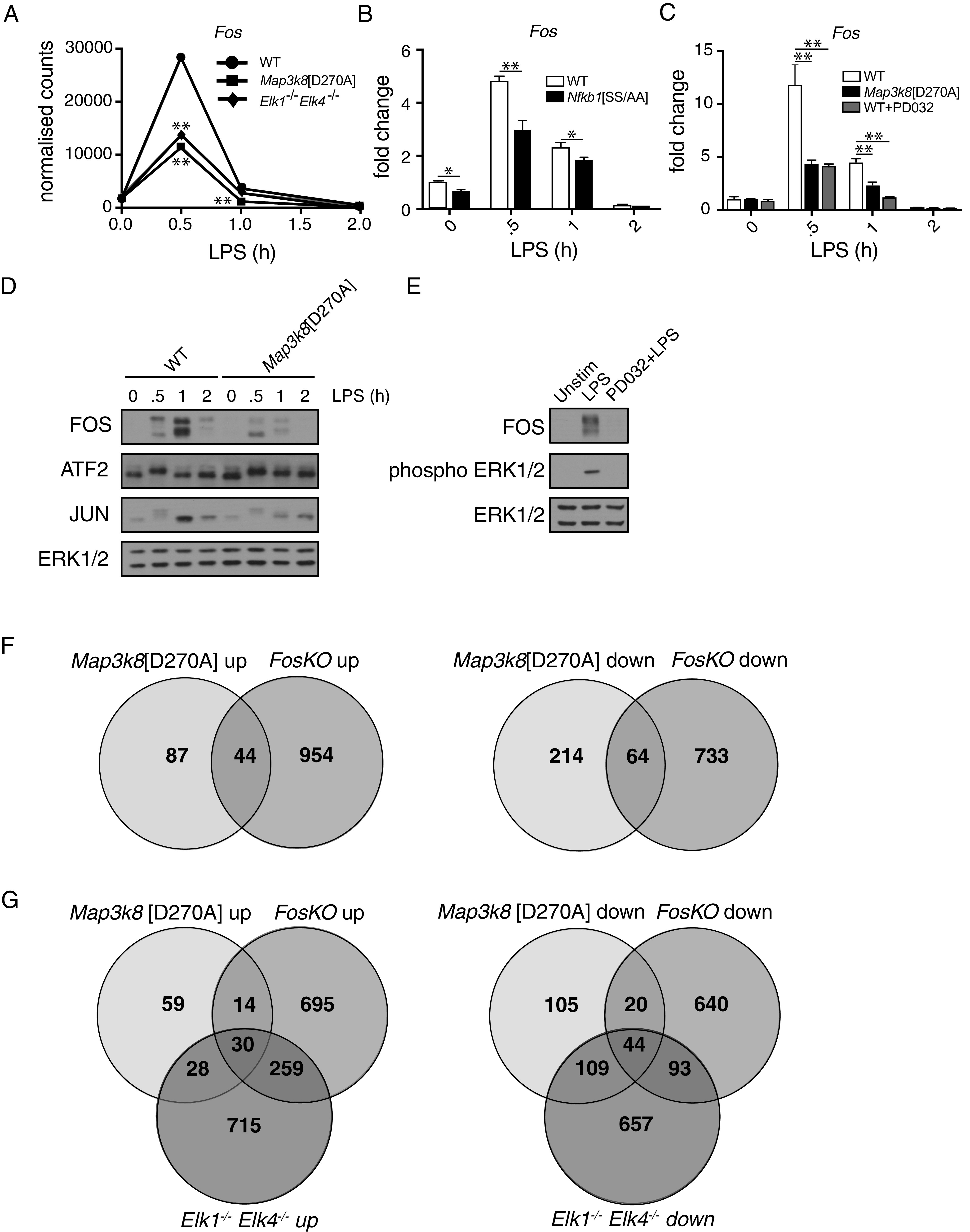
TPL-2 controls LPS-induced gene expression via regulation of FOS abundance. (**A**) Normalized counts for *Fos* from the RNA-seq analyses of WT, *Map3k8*[D270A], and *Elk1*^−/−^*Elk4*^−/−^ BMDMs stimulated with LPS for indicated times. *p ≤ 0.01. (**B**) WT and *Nfkb1*[SS/AA] BMDMs were stimulated with LPS during a 2-h time course. *p ≤ 0.01, **p ≤ 0.01. (**C**) WT BMDMs with and without PD0325901 and *Map3k8*[D270A] BMDMs were stimulated with LPS for the indicated times. In (B) and (C), *Fos* mRNA expression was analyzed by qRT-PCR. Results are representative of three similar experiments. **p ≤ 0.01. (**D**) WT and *Map3k8*[D270A] BMDMs were stimulated with LPS for indicated times. Total cell lysates were immunoblotted for antigens shown. (**E**) WT BMDMs with and without PD0325901 were stimulated with LPS for 30 min. Total cell lysates were immunoblotted for antigens shown. Results in (D) and (E) are representative of three similar experiments. (**F**) Venn diagrams to overlaps of genes upregulated or downregulated (>2-fold) by *Map3k8*[D270A] mutation and FOS deficiency, calculated from *Map3k8*[D270A]/WT and *Fos*KO/LysM-Cre RNA-seq comparisons. (**G**) Venn diagram comparison of TPL-2-, TCF-, and FOS-regulated genes identified in RNA-seq experiments. Overlap of genes upregulated or downregulated by *Map3k8*^D270A^ mutation, TCF deficiency, and FOS deficiency.

Analysis of the *Elk1*^−/−^/*Elk4*^−/−^/WT RNA-seq dataset revealed that TCF deficiency suppressed the expression of all *Fos* family gene mRNAs following LPS stimulation of macrophages in a similar fashion to *Map3k8*^D270A^ mutation (Fig. 4A, Supplemental Figs. 4C, 5A). The pronounced effects on the expression of *Fos* genes suggested that TCF control of gene expression in LPS-stimulated macrophages was mediated via control of *Fos* family transcription. Because *Fos* is a known direct target of TCF-mediated transcription (42) and *Fos* mRNA was expressed at much higher levels in LPS-stimulated macrophages than *FosB*, *Fra1*, and *Fra2* (Supplemental Fig. 4C), we focused on the role of FOS in mediating TPL-2 regulation of gene expression.

qRT-PCR demonstrated that LPS-induced expression of *Fos* mRNA in macrophages was significantly reduced by both *Map3k8*^D270A^ and *Nfkb1*^SSAA^ mutations (Fig. 4B, 4C). PD0325901 treatment also significantly reduced *Fos* mRNA levels following LPS stimulation of WT macrophages (Fig. 4C). Immunoblotting experiments confirmed that LPS-induced expression of FOS protein was reduced by *Map3k8*^D270A^ mutation (Fig. 4D) and pharmacological blockade of ERK1/2 activation in WT cells with PD0325901 (Fig. 4E). Taken together, these results indicated that TPL-2 controlled *Fos* mRNA and FOS protein expression via an IKK2–NF-κB1 p105–TPL-2–ERK1/2 MAPK signaling pathway. However, *Map3k8*^D270A^ mutation only partially blocked LPS induction of *Fos* mRNA (Fig. 4C) and FOS protein (Fig. 4D), indicating that LPS-induced *Fos* transcription was only partially dependent on TPL-2–ERK1/2 signaling.

To determine the contribution of FOS to LPS-induced gene expression, BMDMs generated from *Fos*^FL/FL^ LysM-Cre (FosKO) and *Fos*^FL/+^ LysM-Cre (LysM-Cre) control mice were stimulated with LPS and gene expression was analyzed by RNA-seq. FOS deficiency significantly reduced LPS-induced *Fra1* and *Fra2* mRNA levels while delaying LPS induction of *FosB* mRNA (Supplemental Fig. 5B). The absence of FOS, therefore, significantly reduced expression of other *Fos* family genes, as previously shown (43, 44).

Genes identified in RNA-seq datasets to be regulated by *Tpl2*^D270A^ mutation or FOS deficiency were compared (Fig. 4F). Of the 131 genes whose expression was increased by *Tpl2*^D270A^ mutation (≥2-fold) during the 2-h LPS time course (including unstimulated), approximately one third (44/131) were also upregulated (≥2-fold) in FosKO BMDMs compared with LysM-Cre cells at any time point. Furthermore, 23% (64/278) of genes whose expression was decreased (≥2-fold) by *Tpl2*^D270A^ mutation were also downregulated (≥2-fold) in FosKO BMDMs compared with LysM-Cre cells. These results indicate that FOS mediates, directly and indirectly, more than a fourth (108/409) of the transcriptional output of TPL-2 signaling in TLR4-stimulated macrophages.

To determine whether TPL-2 regulates gene expression via a TCF-FOS signaling axis in LPS-stimulated macrophages, genes modulated by *Tpl2^D270A^* mutation were compared with genes modulated by TCF deficiency and FOS deficiency (Fig. 4G). Twenty-nine percent (44/153) of the genes that were downregulated both in *Tpl2^D270A/D270A^* and *Elk1/4*^−/−^ cells relative to WT cells were also downregulated in FosKO BMDMs compared with LysM-Cre controls. In addition, >50% (30/58) of genes upregulated in both *Tpl2^D270A/D270A^* and *Elk1/4*^−/−^ BMDM RNA-seq datasets were also upregulated in FOS-deficient cells. These results indicate that a significant fraction of genes regulated by TPL-2 catalytic activity in LPS-stimulated macrophages were controlled via FOS by TCF-mediated *Fos* transcription. LPS stimulation induced phosphorylation of FOS on two ERK1/2-regulated sites (Ser^362^/Ser^374^) (data not shown), suggesting that TPL-2 may have also regulated FOS levels via ERK1/2 regulation of FOS protein stability (45).

### Inhibition of type I IFN expression by the TPL-2–TCF–FOS signaling pathway

To calculate enrichment of FOS-regulated genes within particular pathways, FOS-regulated genes at 0.5 h of LPS stimulation were analyzed using the MetaCore program. The three most significantly enriched pathways among genes upregulated by FOS deficiency at 0.5 h were all linked to type I IFN signaling (Supplemental Fig. 5C). Gene set enrichment analysis also identified type I IFN signaling as the most enriched pathway among TPL-2–regulated cluster 4 genes (data not shown), in which several FOS family members were identified as potential transcription factors.

**FIGURE 5.**
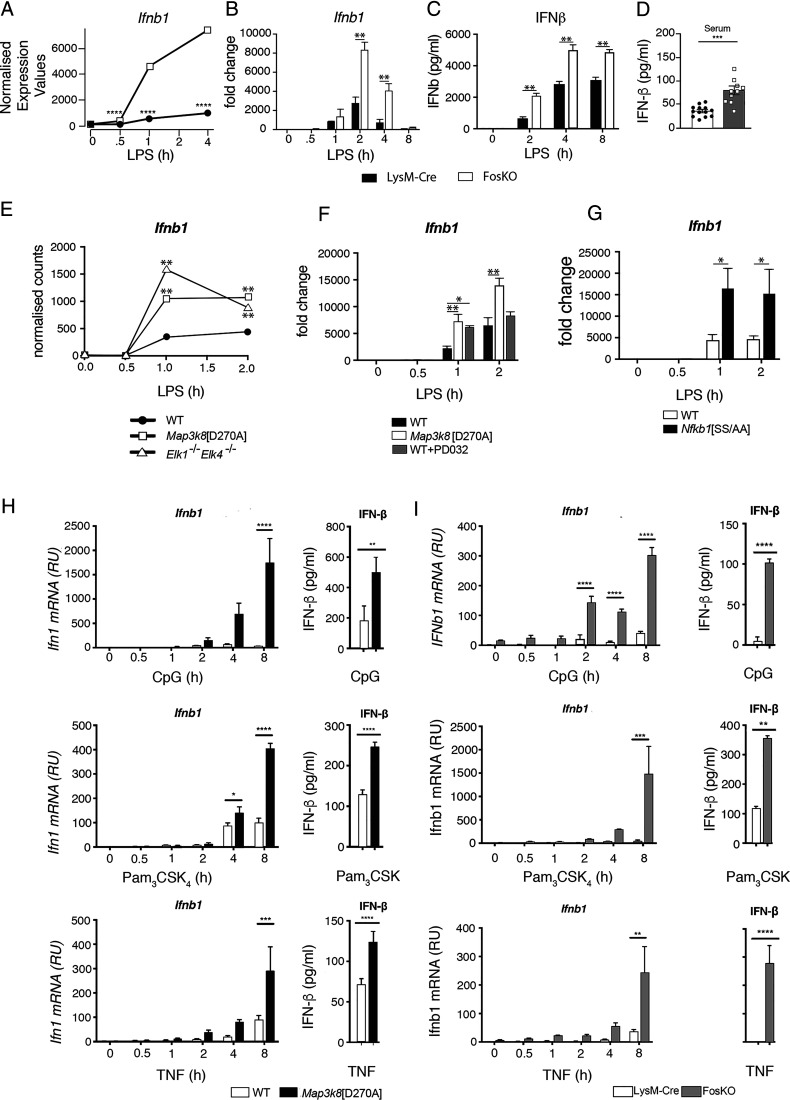
FOS represses LPS-induced expression of IFN-β. (**A**) Normalized counts for *Ifnb1* from the RNA-seq analysis of *Fos*KO and LysM-Cre BMDMs stimulated with LPS for the indicated times. ****p ≤ 0.0001. (**B** and **C**) *Fos*KO and LysM-Cre BMDMs were stimulated with LPS during an 8-h time course. In (B), *Ifnb1* mRNA expression was analyzed by qRT-PCR. In (C), secreted IFN-β in culture supernatants was assayed by ELISA. Results are representative of three similar experiments. **p ≤ 0.01. (**D**) FosKO and control LysM-Cre mice were injected i.p. with LPS. IFN-β in serum at 4 h was assayed by ELISA. Data are pooled from three independent experiments. Mean ± SEM. ***p < 0.001. One-way ANOVA. (**E**) Normalized counts for *Ifnb1* from the RNA-seq analysis of WT, *Map3k8*[D270A], and *Elk1*^−/−^*Elk4*^−/−^ BMDMs stimulated with LPS for indicated times. (**F**) WT BMDMs with and without PD0325901 and *Map3k8*[D270A] macrophages were stimulated with LPS for a 2-h time course. *Ifnb1* expression was analyzed by qRT-PCR. (**G**) WT and *Nfkb1*[SS/AA] BMDMs were stimulated with LPS for the indicated times. *Ifnb1* expression was analyzed by qRT-PCR. (**H**) WT and *Map3k8*[D270A] BMDMs were stimulated for the indicated times with CpG, Pam_3_CSK_4_, or TNF. *Ifnb1* mRNA levels were determined by qRT-PCR (left graphs). IFN-β in culture supernatants after 24-h cultures was determined by ELISA (right graphs). (**I**) LysM-Cre control and *Fos*KO BMDMs were stimulated for the indicated times with CpG, Pam_3_CSK_4_, or TNF. *Ifnb1* mRNA levels were determined by qRT-PCR (left graphs). IFN-β in culture supernatants after 24-h cultures was determined by ELISA (right graphs). Results in (B)–(E) are representative of three similar experiments. For (E)–(I), *p ≤ 0.05, **p ≤ 0.01, ***p ≤ 0.001, ****p ≤ 0.0001.

Analysis of the FosKO/LysM-Cre RNA-seq dataset revealed that FOS deficiency significantly augmented LPS induction of *Ifnb1* mRNA (Fig. 5A). qRT-PCR confirmed that FOS deficiency augmented *Ifnb1* mRNA expression after LPS stimulation of BMDMs (Fig. 5B), with increased IFN-β protein detected in culture supernatants (Fig. 5C). Serum levels of IFN-β protein were also significantly increased in FosKO mice compared LysM-Cre controls after LPS injection, confirming the physiological relevance of the in vitro findings. mRNA levels of multiple IFN-stimulated genes (ISGs) (46) were elevated at 2 h of LPS in *Fos*KO BMDMs compared with control LysM-Cre control cells (Supplemental Fig. 5D). Furthermore, mRNA levels of *Ifna2*, *Ifna4*, *Ifna5*, *Ifna6*, and *Infa13*, which were not induced by LPS in control cells, were increased following LPS stimulation of FosKO BMDMs (Supplemental Fig. 6). These results indicated that FOS deficiency induced a type I IFN signature in LPS-stimulated macrophages by promoting the expression of type I IFNs. The expression of a subset of ISGs was increased by FOS deficiency in unstimulated cells (Supplemental Fig. 5D), possibly suggesting direct suppression of specific ISG expression by FOS in the absence of type I IFN production.

Consistent with our earlier observations with *Tpl2*^−/−^ BMDMs (20), analysis of *Map3k8*^D270A^/WT RNA-seq data demonstrated that *Map3k8^D270A^* mutation increased *Ifnb1* mRNA levels in BMDMs following LPS stimulation (Fig. 5E). qRT-PCR quantitation confirmed this finding and also showed that LPS-induced expression of *Ifnb1* mRNA was increased by PD0325901 treatment of WT BMDMs and by *Nfkb1*^SSAA^ mutation (Fig. 5F, 5G). Furthermore, TCF deficiency (*Elk1/4*^−/−^/WT BMDM RNA-seq comparison) significantly increased *Ifnb1* mRNA after LPS stimulation (Fig. 5E). Taken together, these results show that TPL-2 repression of *Ifnb1* mRNA expression in LPS-stimulated macrophages is mediated, at least in part, via TCF-induced *Fos* transcription.

To determine the broader significance of TPL-2 and FOS in regulating *Ifnb1* mRNA expression in innate immune responses, BMDMs were stimulated with CpG (TLR9), Pam_3_CSK_4_ (TLR1/2), or TNF. *Map3k8^D270A^* mutation reduced *Fos* mRNA expression induced by each of these ligands (data not shown) and increased both *Ifnb1* mRNA and IFN-β levels (Fig. 5H). *Ifnb1* mRNA expression and IFN-β protein secretion were also significantly increased by FOS deficiency following stimulation of BMDMs with each of the ligands (Fig. 5I). These results indicate that the TPL-2 stimulation of FOS expression plays a general role in suppressing type I IFN signaling in innate immune responses of macrophages.

### TPL-2 regulates IFN-β transcription via multiple pathways

**FIGURE 6.**
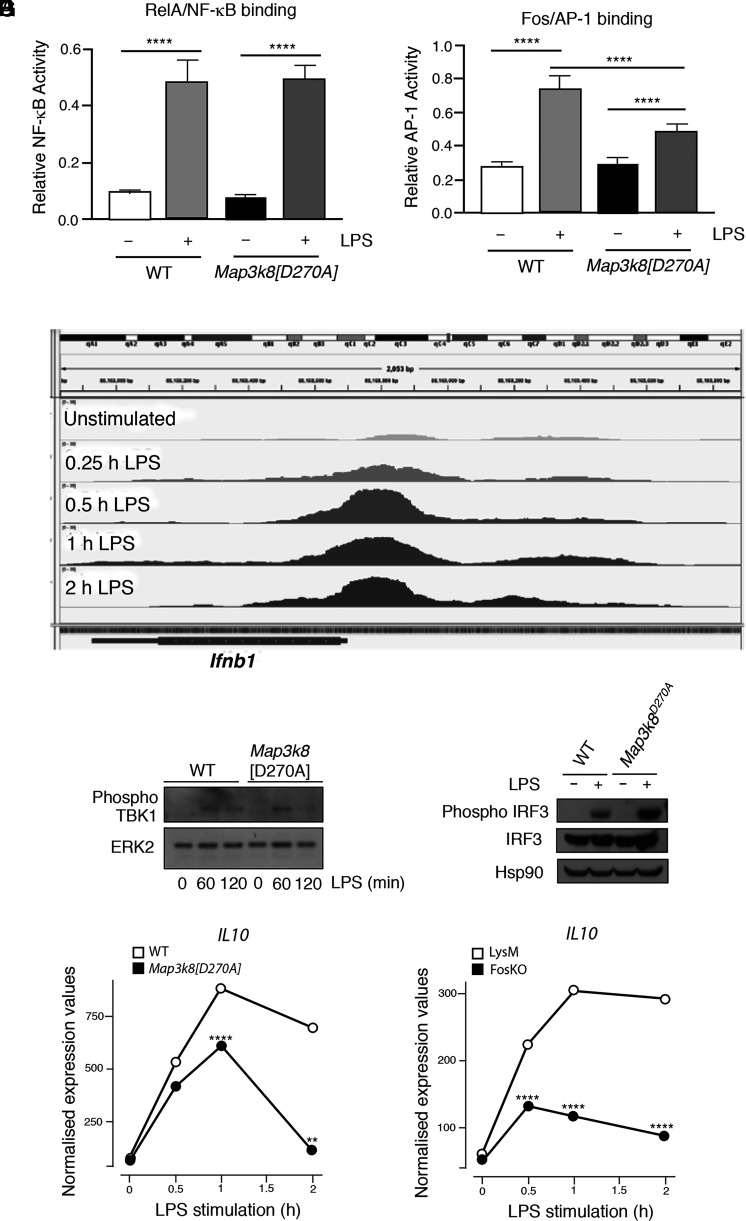
TPL-2 regulation of *Ifnb1* mRNA expression via multiple pathways. (**A** and **B**) WT and *Map3k8*[D270A] BMDMs were stimulated with LPS (30 min). Nuclear extracts were assayed by ELISA for RelA binding to NF-κB and FOS binding to AP-1. Mean of separate cultures from four biological replicates shown ± SEM. ****p < 0.0001. Two-way ANOVA. Representative of three experiments with similar results. (**C**) ChIP-seq data from Tong et al. (46) of FOS binding to *Ifnb1* gene in BMDMs stimulated with LPS during a time course. The figure is an annotated screenshot of the Integrative Genomics Viewer output at the *Ifnb1* gene location within the mm10 genome. (**D** and **E**) WT and *Map3k8*[D270A] BMDMs were stimulated with LPS at the indicated times in (D) and for 60 min in (E). Total lysates were immunoblotted for the indicated antigens. (**F**) Normalized counts from the RNA-seq analysis of WT and *Map3k8*[D270A] macrophages unstimulated or stimulated with LPS for the indicated times are shown *Il10*. **p ≤ 0.01, ****p ≤ 0.0001. (**G**) Normalized counts for *Il10* from the RNA-seq analysis of *Fos*KO and LysM-Cre BMDMs stimulated with LPS for the indicated times. ****p ≤ 0.0001.

IFN-β transcription is regulated by the cooperative binding of ATF2-JUN AP-1, IRF3/IRF7, and NF-κB to the *Ifnb1* enhanceosome (47). An NF-κB ELISA demonstrated that *Map3k8*^D270A^ mutation inhibited *Ifnb1* transcription independently of effects on NF-κB activation following LPS stimulation of BMDMs (Fig. 6A). LPS-induced FOS/AP-1 binding was reduced by *Map3k8*^D270A^ mutation, as expected (Fig. 6B), while analysis of published ChIP/sequencing data (46) showed that FOS is recruited to the *Ifnb1* gene following LPS stimulation of BMDMs (Fig. 6C). This suggested that FOS-containing AP-1 dimers may directly inhibit *Ifnb1* transcription.

IRF3 is activated by phosphorylation by TBK1 (48). Immunoblotting demonstrated that LPS induction of TBK1 Ser^172^ (activating) phosphorylation was not affected by *Map3k8*^D270A^ mutation (Fig. 6D). However, IRF3 Ser^385^ phosphorylation was increased in *Map3k8*^D270A/D270A^ BMDMs compared with WT controls (Fig. 6E). These data indicated that TPL-2 signaling inhibits TBK1 phosphorylation of IRF3 to suppress *Ifnb1* transcription following LPS stimulation of macrophages.

The TPL-2–ERK1/2 signaling pathway (49) and FOS (50) suppress LPS induction of the anti-inflammatory cytokine IL-10. IL-10 has profound inhibitory effects on cytokine production by TLR-stimulated macrophages (51). We have previously shown that increased IFN-β production by *Mapk3k8*^−/−^ BMDMs results in part from reduced IL-10 production (20). *Map3k8*^D270A^ mutation (Fig. 6F) and FOS deficiency (Fig. 6G) also significantly decreased expression of *Il10* mRNA induced by LPS stimulation of BMDMs. Reduced IL-10 likely contributed to the increased IFN-β production caused by *Map3k8*^D270A^ mutation and FOS deficiency.

Collectively, the results presented in this section suggest that the mechanism by which TPL-2 suppresses IFN-β production by TLR4-stimulated macrophages involves multiple interconnected pathways, which likely converge to ensure a robust control of cytokine production in response to pathogens.

## Discussion

In this study, RNA-seq was used to comprehensively identify genes regulated by TPL-2 catalytic activity in LPS-stimulated macrophages. Bioinformatics analysis of these genes using the MetaCore pathway analysis tool confirmed the importance of TPL-2 in regulating immune responses and inflammation. Our previous analyses of the TPL-2 phosphoproteome demonstrated that ∼85% of protein phosphorylations regulated by TPL-2 catalytic activity are dependent on ERK1/2 activation (10). Consistent with this, RNA-seq analyses in this study revealed a strong correlation between the effect of an MKK1/2 inhibitor on WT cells and *Map3k8*^D270A^ mutation, indicating that most TPL-2–regulated changes in transcription in response to TLR4 activation were mediated via ERK1/2 activation. However, analysis of gene expression in WT cells treated with the specific RSK inhibitor RSKi47 did not suggest that ERK1/2 activation of RSK had a dominant role in mediating gene expression changes downstream of TPL-2, but rather that ERK1/2 activation itself was important.

Previous studies have focused on regulation of cytokine and chemokine expression by TPL-2 (13, 52–54). Notably, our work demonstrated that TPL-2 signaling also directly controls the expression of several key transcription factors, including EGR1, EGR3, NR4A1, IER3, and FOS, which is likely to have wide-ranging effects on macrophage function. For example, IER3 is known to play a role in inflammatory bowel disease and following *Leishmania* infection (55, 56), whereas NR4A1 modulates apoptosis of monocytes (57). TPL-2 also controlled the expression of genes involved in negative feedback regulation of MAPK signaling (e.g., *Dusp1*, *Dusp2*, and *Dusp5*), which have key roles in controlling inflammation (58, 59). In addition, TPL-2 regulated enzymes controlling extracellular matrix remodeling and PG synthesis, which are important in immune responses (60, 61).

Bioinformatics analysis of cluster 1 genes that contained the genes most rapidly regulated by TPL-2 catalytic activity suggested that the TCF family of transcription factors were involved in controlling their transcription. Consistent with this, phosphorylation of ELK1 on a known ERK1/2 target site was inhibited by *Map3k8*^D270A^ mutation (62). There was also a strong correlation between genes dependent on TPL-2 catalytic activity and those dependent on ELK1/4 expression. Around 50% of the genes downregulated by *Map3k8*^D270A^ mutation between 0.5 and 2 h after LPS stimulation were similarly reduced by deficiency of ELK1 and ELK4 following LPS stimulation of macrophages. Furthermore, of the genes that were upregulated in *Map3k8*^D270A/D270A^ macrophages, ∼40% were similarly upregulated in *Elk1*^−/−^ *Elk4*^−/−^ macrophages. This demonstrated that TCFs mediate a substantial fraction of TPL-2–dependent transcriptional output, either directly or via TCF-dependent transcription of downstream transcription factors.

MetaCore analysis of cluster 3 genes identified LITAF (LPS-induced TNF-α factor) (63) as the most enriched transcription factor regulated by TPL-2 catalytic activity. Published work has shown that LITAF forms a complex with STAT6 (64) and is required for LPS induction of cytokines, including TNF-α and IL-6, in macrophages (65). LITAF nuclear translocation is regulated by p38α (65), and the current study raises the possibility that LITAF nuclear translocation and transcription may also be regulated by TPL-2–induced activation of ERK1/2. This will be an important area of research for the future to determine how ERK1/2 signaling controls innate immune responses.

Bioinformatics analysis of rapidly induced TPL-2–regulated genes in LPS-stimulated macrophages suggested an important role for AP-1 transcription factors in mediating the transcriptional output of TPL-2. AP-1 factors cooperate with other DNA binding proteins to promote transcription, and a recent ChIP-seq analysis indicated that adjacent NF-κB binding motifs are highly correlated with binding of all AP-1 monomers in TLR4-stimulated macrophages (66). Furthermore, there was a statistically significant enrichment of NF-κB target genes, identified by RelA binding within 2 kbp of the transcriptional start site (67), among those genes regulated by TPL-2 catalytic activity in LPS-stimulated BMDMs (data not shown). The coordinated activation of AP-1 with the activation of NF-κB transcription factors via IKK-mediated control of TPL-2 signaling (7) may ensure efficient expression of AP-1/NF-κB target genes in TLR-stimulated macrophages.

Similar to *Map3k8*^D270A/D270A^ macrophages, ELK1/ELK4-deficient macrophages have elevated levels of *Ifnb1* mRNA compared with WT macrophages following TLR4 stimulation. We have previously shown that FOS overexpression reduces TLR induction of *Ifnb1* mRNA in *Map3k8*^−/−^ DCs, suggesting an inhibitory role for FOS in *Ifnb1* transcription (20). Consistent with this, RNA-seq revealed that *Ifnb1* mRNA levels were significantly elevated in LPS-stimulated FOS-deficient macrophages compared with controls, and type I IFN signature pathways were significantly enriched among genes whose expression was increased by the absence of FOS. Furthermore, FOS deficiency facilitated LPS upregulation of IFN-α isoforms in macrophages (which was not evident in LPS-stimulated WT macrophages) and the expression of multiple ISGs was augmented. Taken together, these results indicate that TPL-2 negatively regulates expression of type I IFN, in part by TCF-induced *Fos* transcription. TPL-2 may additionally control FOS protein stability via ERK1/2-mediated FOS phosphorylation, similar to fibroblasts (45).

*Ifnb1* transcription involves the coordinated binding of ATF2-JUN AP-1 dimers, IRF3, and NF-κB to the *Ifnb1* enhanceosome (47). *Map3k8*^D270A^ mutation did not affect LPS activation of NF-κB, although FOS binding to AP-1 was significantly reduced. Analysis of published ChIP sequencing data (46) revealed that LPS stimulation of macrophages induces the recruitment of FOS to the *Ifnb1* gene, suggesting that TPL-2 induction of FOS may directly inhibit *Ifnb1* transcription. We have previously shown that PPARγ transcription can be positively or negatively regulated by AP-1, depending on the composition of the AP-1 dimer (68). The present study suggests that TPL-2 signaling reduces *Ifnb1* transcription by controlling the abundance of FOS, which can form inhibitory AP-1 dimers that compete with activating ATF2-containing AP-1 dimers. TPL-2 signaling additionally inhibits IRF3 phosphorylation by TBK1, which will directly reduce *Ifnb1* transcription. TPL-2 induction of IL-10 expression via FOS also contributes to the suppression of type I IFN expression by TPL-2 (20, 50). TPL-2 signaling, therefore, appears to inhibit type I IFN expression via multiple complementary mechanisms.

FOS-dependent repression of *Ifnb1* expression in macrophages was not restricted to LPS stimulation. Other TLR agonists (CpG and Pam_3_CSK_4_) and TNF suppressed *Ifnb1* mRNA levels in macrophages via TPL-2–induced FOS expression. Furthermore, LPS- and TNF-stimulated increases in *Ifnb1* mRNA in fibroblasts derived from mice lacking Elk1, Elk3, and Elk4 were increased relative to WT controls (data not shown). The repression of *Ifnb1* transcription by TPL-2 regulation of FOS levels therefore may be of general importance in innate immune responses in myeloid and stromal cells.

It is well established that TCFs regulate proliferation of fibroblasts following growth factor stimulation (23, 24) and also control T cell development and function (27). However, a role for TCFs in innate immunity has not been established. The present study identified TCFs and their downstream target FOS as major mediators of TPL-2 transcriptional output in the innate immune response of macrophages, which is particularly important for suppressing the production of type I IFNs. Our earlier experiments have shown that TPL-2 negative regulation of type I IFN signaling is essential for efficient innate immune responses to MTB and *L. monocytogenes* (21). The present study strongly supports a role for TPL-2 signaling in promoting the innate immune responses to these intracellular bacteria by inducing TCF-dependent *Fos* transcription to inhibit macrophage production of IFN-β. Type I IFNs also negatively regulate immune responses to *Salmonella* Typhimurium (69). Furthermore, immune responses to *S.* Typhimurium are dependent on FOS expression (70). RNA-seq has demonstrated that *Map3k8*^D270A^ mutation significantly reduces *Fos* mRNA upregulation while increasing *Ifnb1* mRNA expression after *S.* Typhimurium infection of BMDMs (L. Blair, F. Breyer, and S.C. Ley, unpublished observations). It will be interesting in future studies to determine whether TPL-2 suppression of type I IFNs is required for efficient innate immune responses to *S.* Typhimurium. In addition, it will be important to evaluate the role of TPL-2 in innate immune responses to pathogenic bacteria that are dependent on type I IFN signaling, such as *Helicobacter pylori* and *Streptococcus pneumoniae* (71).

## Supplementary Material

Data Supplement
