# Supplementary material for: TPL-2 Inhibits IFN-β Expression via an ERK1/2-TCF-FOS Axis in TLR4-Stimulated Macrophages
Source: J Immunol. 2022 Feb 15;208(4):941–54. doi: 10.4049/jimmunol.2100213 (PMC9012084; doi:10.4049/jimmunol.2100213)
Supplement: Data Supplement [file JI_2100213.zip › JI_2100213_Supplemental_1.pdf]

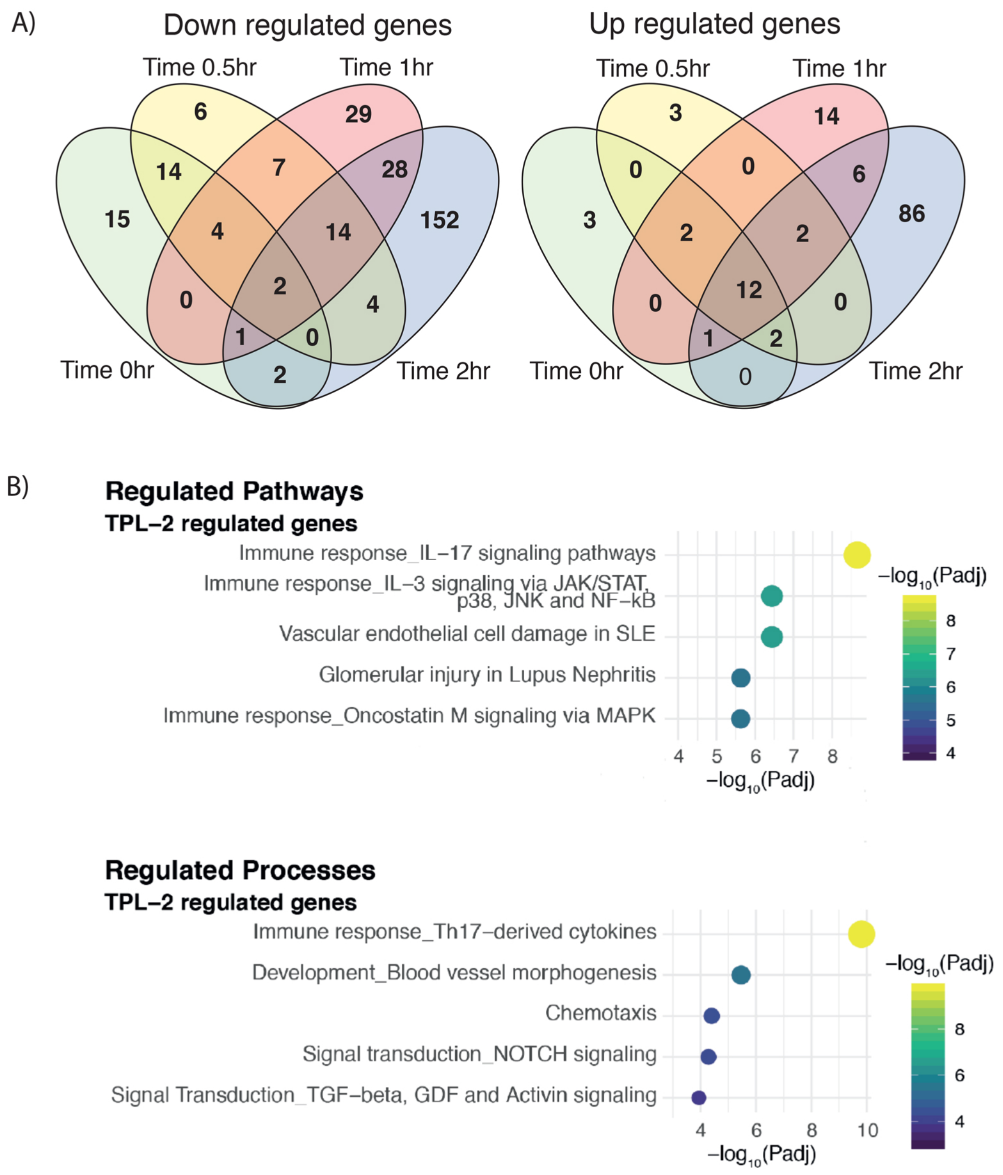

Supplementary figure 1

Supplementary Figure 1

TPL-2 regulates genes involved in immune responses in LPS-stimulated macrophages

A) WT and *Map3k8*[D270A] macrophages were stimulated with LPS for the indicated times and gene expression was determined by RNAseq. Venn diagram shows overlaps of genes upregulated or downregulated (>2-fold) by *Map3k8*[D270A] mutation over the time course.

B) Metacore analysis of the 391 genes found to be regulated (up or down) by TPL-2 kinase activity by 2-fold in the RNAseq dataset. Most enriched pathways (top panel) or processes (bottom panel) are presented, ranked by -log10(p adjusted (Padj) value), size and colour of data points were also proportional to -log10(Padj).

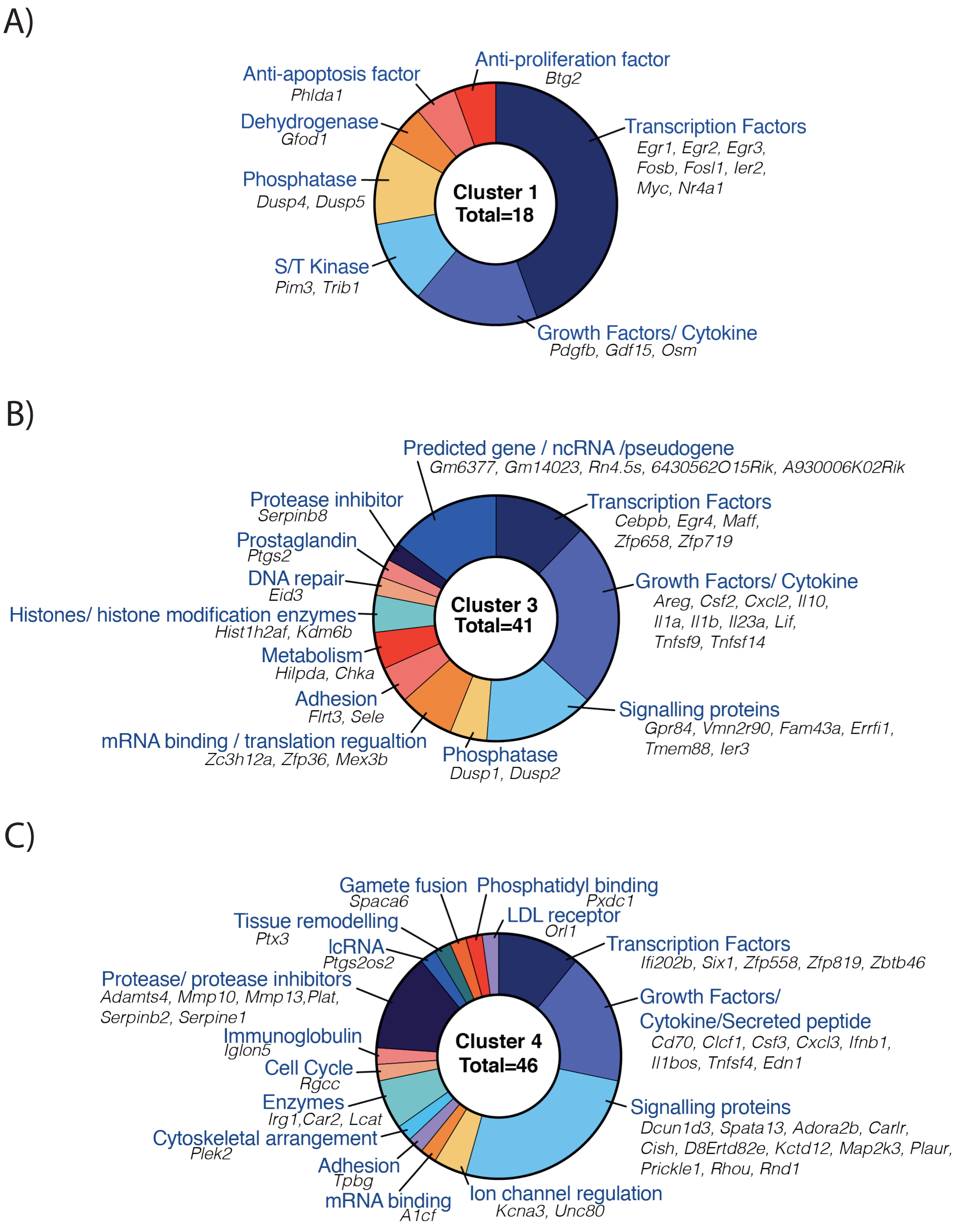

Supplementary figure 2

Supplementary Figure 2

Functional classification of genes rapidly induced by TPL-2 signaling in LPS-stimulated macrophages

Genes regulated by TPL-2 catalytic activity were identified by K-mean analysis of RNAseq data from LPS-stimulated WT and *Map3k8*[D270A] BMDMs (see Figure 1B). The genes in cluster 1 (A), cluster 3 (B) and cluster 4 (C) were classified by function using gene ontology, Uniprot and literature review.

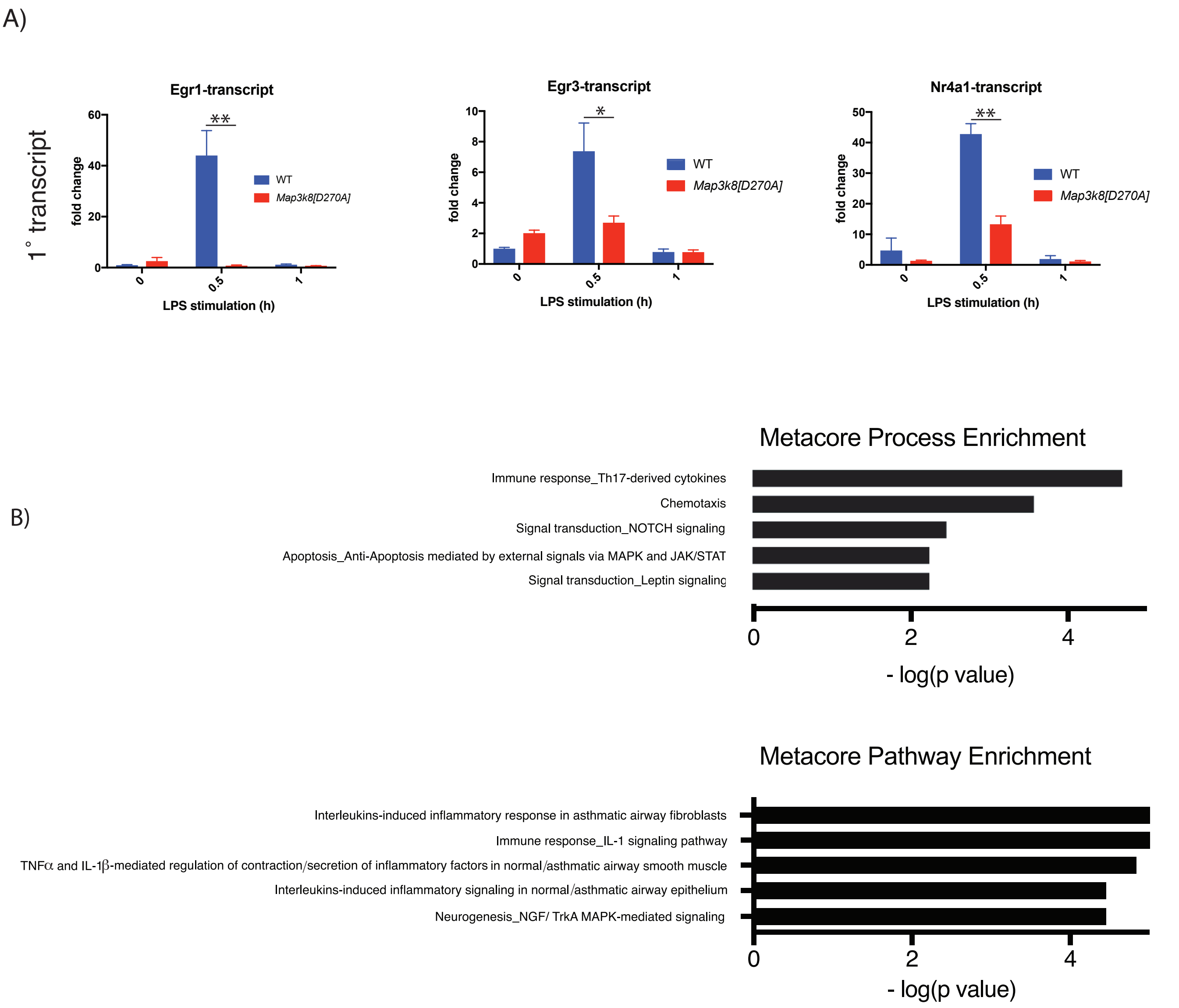

Supplementary Figure 3

Supplementary Figure 3

TPL-2 and TCFs co-regulate immediate early genes and genes involved in immune responses in LPS-stimulated macrophages

A) WT and *Map3k8*[D270A] macrophages stimulated with LPS for a 2 hour timecourse. Total RNA was extracted and *Egr1*, *Egr3* and *Nr4a1* primary transcript levels analysed by qRT-PCR. Results are representative of three similar experiments. * p≤0.05; ** p≤0.01.

B) Metacore analysis of the genes co-regulated (>2 fold) by TPL-2 and TCFs in LPS-stimulated macrophages, as identified by RNAseq analyses of WT/*Map3k8*[D270A] and WT/*Elk1*$^{-/-}$*Elk4*$^{-/-}$ BMDMs (see Figure 3). Most enriched pathways and processes are presented, ranked by log(p value).

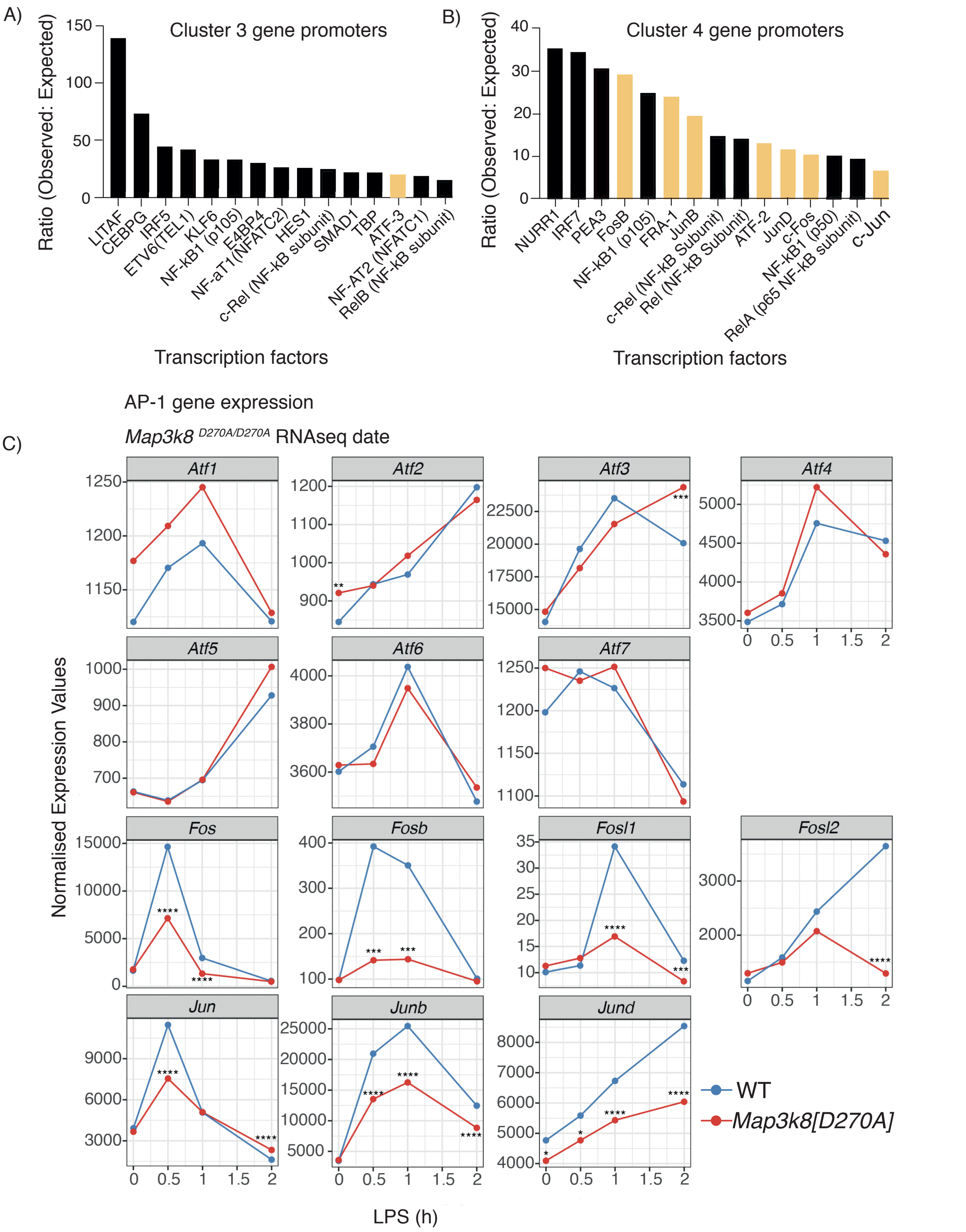

Supplementary Figure 4

Supplementary Figure 4

Bioinformatic analysis identifies AP-1 transcription factors as potential mediators of TPL-2 regulated gene expression in LPS-stimulated macrophages

Genes in cluster 2 (A) and cluster 4 (B) were analysed for transcription factor enrichment using the Metacore programme. The top 15 enriched transcription factors are shown ranked by the ratio of observed sites to expected sites in the cluster 1 gene promoters. AP-1 family members are shown in yellow. C) Normalised counts for indicated AP-1 family genes from the RNAseq analyses of WT and *Map3k8*[D270A] stimulated with LPS for indicated times. **p≤0.01; *** p≤0.001; **** p≤0.0001.

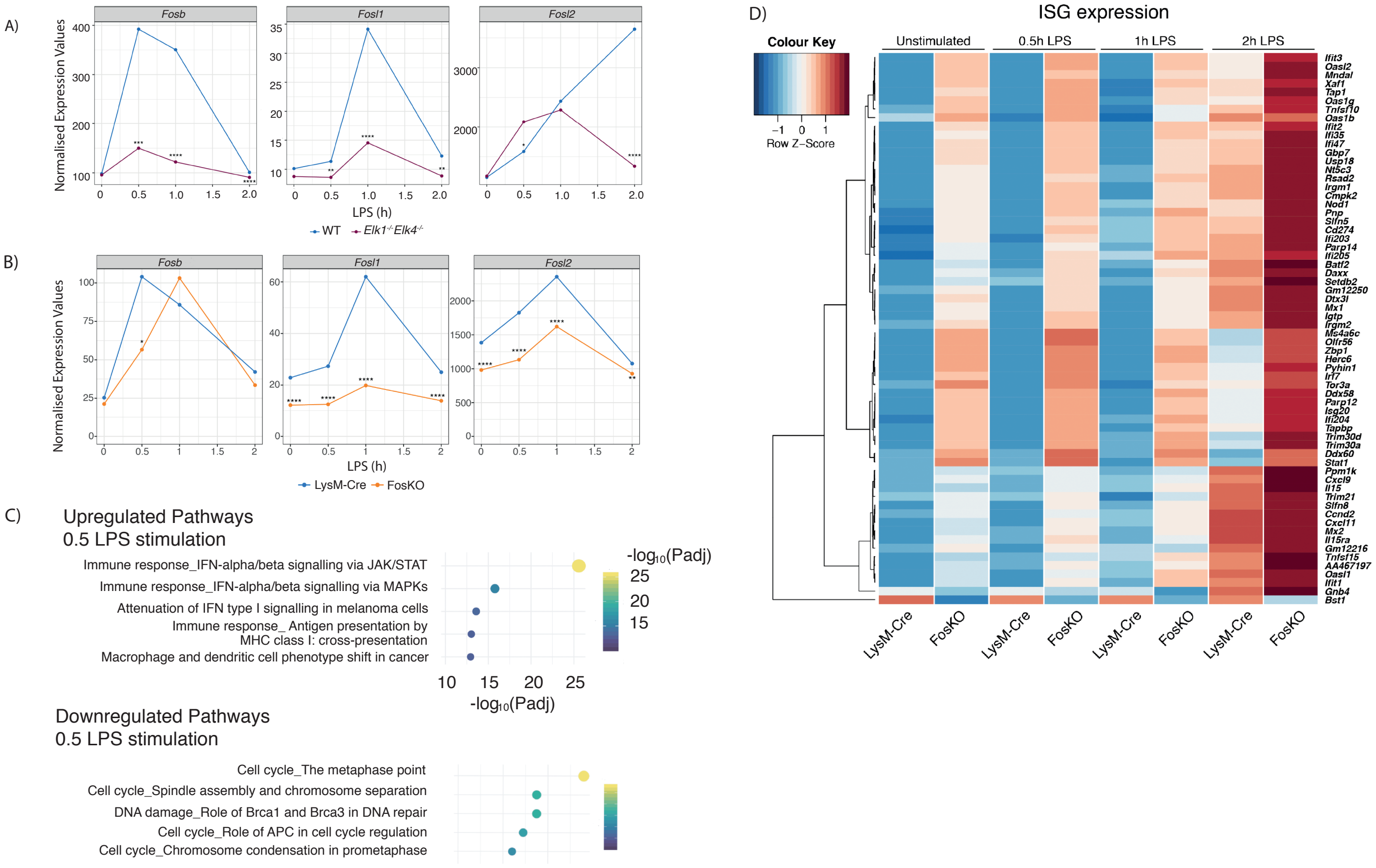

Supplementary Figure 5

Supplementary Figure 5

FOS-deficiency upregulates type I interferon pathways in LPS-stimulated macrophages

A and B) Normalised counts from the RNAseq analysis of (A) WT and *Elk1*$^{-/-}$*Elk4*$^{-/-}$ macrophages and (B) FosKO and LysM-Cre (control) macrophages, unstimulated or stimulated with LPS hours for the indicated Fos family genes. * p≤0.05; ** p≤0.01; *** p≤0.001; **** p≤0.00001. C)The top 5 most enriched pathways are presented, ranked by -log10(p adjusted (Padj) value); size and colour of data points are also proportional to -log10(Padj). (Top panel) Metacore analysis of genes upregulated in FosKO BMDMs compared to WT at 0.5 h LPS stimulation. (Bottom panel) Metacore analysis of genes downregulated in FosKO BMDMs compared to WT at 0.5 h LPS stimulation. D)Heatmap generated from RNAseq showing row normalised expression for ISGs (39) in LysM-Cre and FosKO macrophages stimulated with LPS for the indicated times.

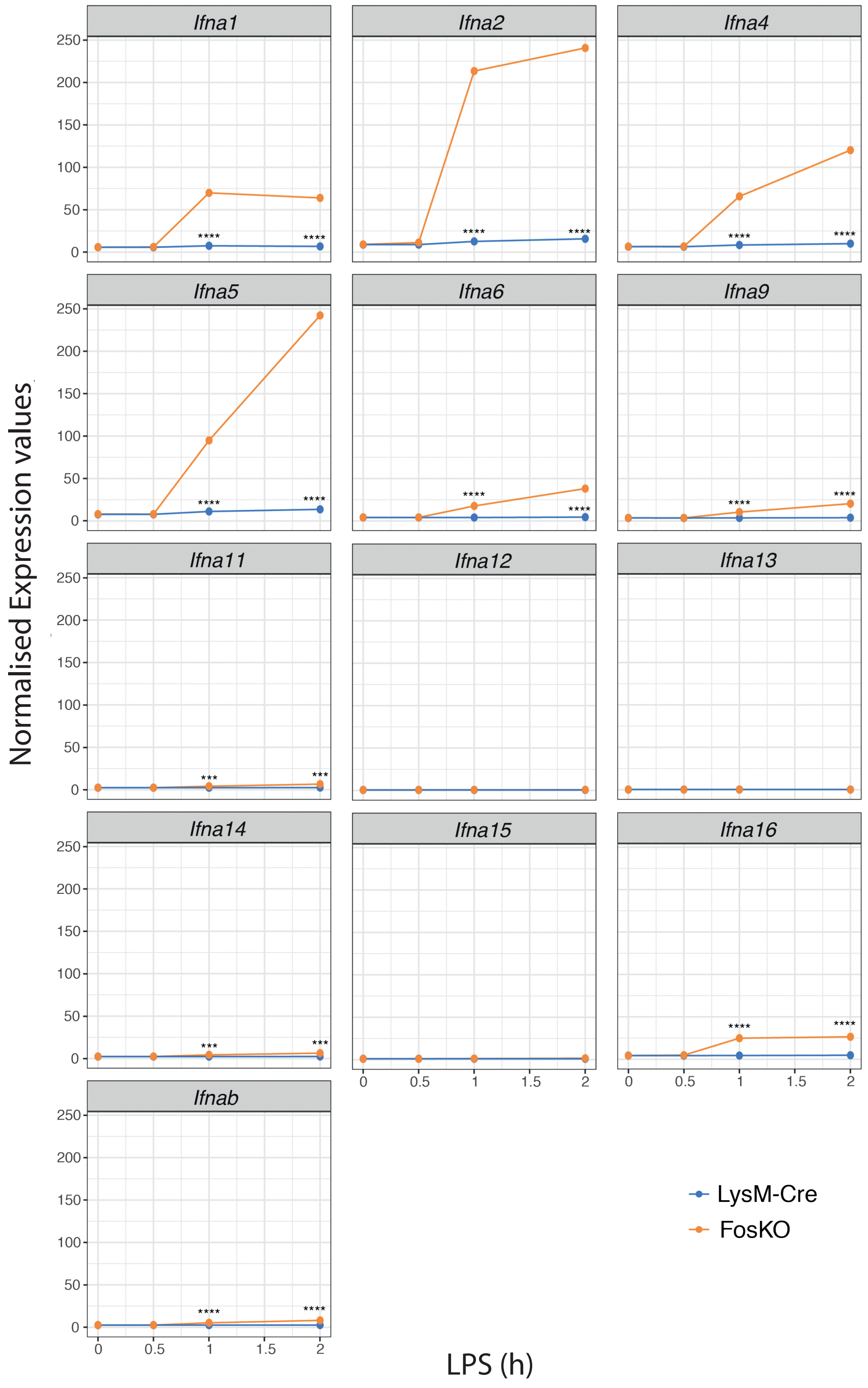

Supplementary Figure 6

Supplementary Figure 6

FOS-deficiency induces expression of alpha interferons in LPS-stimulated macrophages

A) Normalised counts from the RNAseq analysis of FosKO and LysM-Cre (control) macrophages, unstimulated or stimulated with LPS hours for the indicated type I IFN genes. *** p≤0.001; **** p≤0.0001.
